# ‘Unheard,’ ‘uncared for’ and ‘unsupported’: The mental health impact of Covid -19 on healthcare workers in KwaZulu-Natal Province, South Africa

**DOI:** 10.1371/journal.pone.0266008

**Published:** 2022-05-04

**Authors:** Bilkis Dawood, Andrew Tomita, Suvira Ramlall

**Affiliations:** 1 Discipline of Psychiatry, School of Clinical Medicine, College of Health Sciences, University of KwaZulu-Natal, Durban, South Africa; 2 KwaZulu-Natal Research Innovation and Sequencing Platform (KRISP), College of Health Sciences, University of KwaZulu-Natal, Durban, South Africa; 3 Centre for Rural Health, School of Nursing and Public Health, College of Health Sciences, University of KwaZulu-Natal, Durban, South Africa; The University of Sydney, AUSTRALIA

## Abstract

As a direct consequence of the Covid-19 pandemic, due to being exposed to chronic and multiple sources of psychological stress, healthcare workers constitute a vulnerable population. Despite the potential impact of Covid-19 on their psychological and physical health, insufficient attention has been given to their mental well-being. The primary objective of this study was to measure and understand this psychological impact on public sector doctors and nurses in KwaZulu-Natal Province, South Africa. The secondary objective was to ascertain their perceptions of psychosocial support, specific to Covid-19, within the workplace. This cross-sectional electronic survey was conducted from August to October 2020, following the first surge of the pandemic in the country, and included 312 participants. Depression, anxiety and stress symptoms were assessed with the Depression Anxiety and Stress Scale-21 item and post-traumatic stress was measured by the Impact of Events Scale-Revised version. Measures of employer support were assessed using an adapted closed-ended questionnaire. The participants’ mean age was 36.6± 9.3 years with three quarters being male (n = 234, 75.0%) and predominantly (n = 214, 72.3%) medical doctors. Numbers of participants with depression, anxiety and stress were 121 (51.5%), 111 (47.2%) and 104 (44.3%) respectively, with 38 (16.2%), 50 (21.3%) and 38 (16.2%) in the combined severe/extremely severe range, respectively. On the Impact of Events Scale-Revised, 34 (13.7%) participants were in the severe range. Subjectively, 63.0% felt that their concerns were not ‘heard’, 75.1% did not feel ‘cared for’ and 81.1% and 74.0% did not feel ‘physically’ or ‘psychologically’ supported, respectively. High levels of depression, anxiety, stress and traumatic stress, combined with poor perceptions of employer support, highlight the need to identify and address the psychosocial support needs and expectations of healthcare workers for the duration of the pandemic, as well as for the mental health sequelae post-pandemic.

## Introduction

On the 30^th^ of January 2020, the World Health Organization (WHO) declared the Covid-19 outbreak a public health emergency of international concern [1]. By the 11^th^ of March 2020, it was declared a global pandemic [2]. The Centres for Disease Control and Prevention (CDC) defines a pandemic as an epidemic that has spread over several countries or continents, usually affecting large numbers of people [3]. This unprecedented pandemic has placed severe physical and psychological pressure upon healthcare workers (HCWs), significantly so in low-income and middle-income countries (LMICs) [4–6].

Healthcare workers have been noted to have an increased susceptibility to the physical risks associated with Covid-19 [7,8]. In the first year of the pandemic in KwaZulu-Natal (KZN), as of the 20^th^ of February 2021, 15 829 HCWs were reported to have been infected with Covid-19 in the public sector, of whom 335 died [9]. In light of this, the physical protection of HCWs has been highlighted as a ‘critical’ part of the Covid-19 response [10]. However, the paradigm of protection largely focuses on physical risks, with decreased attention addressing the concomitant psychological risks inherent in frontline healthcare work during the pandemic, particularly in LMICs [6]. Biologically, chronic stress has been reported to have adverse physiological effects, including hormonal, immunological and cognitive functioning [11–13]. Depression, anxiety and stress (DAS) and post-traumatic stress disorder (PTSD) have been shown to be associated with immune and inflammatory dysregulation mechanisms, notably, responses to viral infections. The pandemic has therefore set in motion a vicious cycle, with a complex interplay of numerous physiological and psychosocial factors. These impact both physical and mental health, and ultimately, the immune system, which is central to the individual impact of and outcome from a viral infection. This highlights that, if healthy outcomes are to be achieved for both the healthcare providers and the patients they serve, a holistic pandemic action plan should address both the obvious biological threat as well as the inherent psychosocial stress. Similarly, the current vaccine rollout highlights physical immunity, but nothing of the need for ‘psychological immunity’ for HCWs who have been exposed to a new and unpredictable situation fraught with emotional and ethical complexities [14,15]. Psychological immunity is defined as “a system of adaptive resources and positive personality characteristics that acts as psychological antibodies at the time of stress” [14]. Beneath this Covid-19 viral pandemic lurks a ‘parallel epidemic’ of deteriorating mental health that requires acknowledgement and necessitates action [16].

Results from studies conducted on Covid-19 to date and previous infectious disease outbreaks, highlight that in these situations HCWs experience high levels of DAS and post-traumatic stress (PTS) symptoms [4,17–22]. Studies conducted during this pandemic have shown that medical HCWs (medical doctors and nurses) have a higher prevalence of mental health-related symptoms than non-medical HCWs [23,24]. A 2020 rapid systematic review of the impact of viral epidemic outbreaks on the mental health of frontline HCWs reported the pooled prevalence for clinically significant symptoms of mental disorders as highest for anxiety, followed by depression and post-traumatic stress disorder (PTSD) [11]. Moreover, after the Severe Acute Respiratory Syndrome (SARS) pandemic in 2012, symptoms of psychological distress were found to persist up to three years after the crisis [25]. One prospective six-month study, conducted in 2020, during the first year of the pandemic, reported a persistence and slight increase of stress and burnout which persisted beyond lockdown periods [22].

A health survey of South African HCWs, conducted during the early part of the Covid-19 pandemic, found poor general health and well-being in 23.7% of participants, while 19.4% to 53.6% reported levels of ‘psychological distress’ [26]. In SA, and particularly in KZN, HCWs represent a vulnerable population, as they are exposed to high work demands in the context of a resource constrained health sector [27]. Mental health services for them may be overshadowed by other local health needs, which include the high burden of infectious diseases, such as the Human Immunodeficiency Virus (HIV) and tuberculosis (TB) [27]. Pre-pandemic, the KZN Department of Health (DOH) had existing resource and infrastructural challenges [28], and burnout, anxiety and depressive symptoms had already been shown to be highly prevalent in HCWs in South Africa [27]. The unprecedented Covid-19 pandemic has thus added a considerable additional burden to a strained and constrained health force.

Psychological distress experienced by HCWs during the pandemic are multifactorial in origin. Personal fear was combined with the inordinate professional demands made in the context of sub-optimum healthcare resources for both patients and HCWs [22,29]. In addition, grieving for personal losses as well as for patients and colleagues, combined with the challenges associated with lockdown all together pose a formidable demand on any individual’s resilience [30]. Notably, this pandemic has intensified pre-existing ‘professional grief’ issues in HCWs, while they are simultaneously encountering ‘personal grief’[31]. Professional grief refers to the grief experienced by HCWs due to the loss of a patient whereas personal grief refers to the grief experienced by HCWs due to the loss of their own loved ones [31]. Moreover, restricted access to psychological support and the performance of funeral and grieving rituals, due to lockdown regulations, can lead to increased rates of pathological grief in HCWs and this increases their risk for various symptoms of psychological distress [31].

Healthcare workers are a vital resource in both the Covid-19 response and the maintenance of essential services and their motivation and empathy are essential to effective and compassionate patient care [32]. Psychological distress among HCWs can negatively job satisfaction, performance and can impact patient care and safety [10,32,33]. This could have negative financial and healthcare repercussions [34]. Psychological distress experienced by HCWs can therefore have far-reaching short-term and long-lasting negative effects on their mental health and overall psycho-social well-being [11,35] as well as on healthcare services.

In response to the pandemic in 2020, the KZN DOH prepared a ‘mental health toolkit’ for HCWs, which can be accessed via their webpage, containing appropriate guidelines and resources to assist them with the psychological impact of the pandemic and links for further information and assistance [36]. Additionally, the KZN Directorate of Mental Health, in collaboration with its EAP, set up a psychosocial support platform at the level of each of its eleven health districts to address HCW stress and distress [36]. While acknowledging these efforts, there is a growing body of evidence to support the need for understanding and measuring the psychological impact of global health crises [32]. However, locally relevant data is limited, and data from African contexts are urgently needed to inform a more comprehensive and holistic response to pandemics [32]. This data should be utilised to inform the development of future studies, policies, improvements and interventions to contribute to better health outcomes and the general well-being of HCWs, patients and communities. Therefore, the study aimed to assess the psychological impact of the Covid-19 pandemic on public sector doctors and nurses in KZN.

## Methods

### Setting, study design, participants and procedure

The first Covid-19 pandemic surge in KZN ‘peaked’ during late July 2020 [37]. At the onset of the pandemic in KZN, specific healthcare facilities were designated as Covid-19 units. However, in the context of a resource-constrained setting and rising infection rates, this plan was no longer feasible, and this resulted in Covid-19 related care being integrated into all existing public healthcare facilities. This study was planned in March 2020, a few days after the outbreak in South Africa, and in August 2020 to October 2020 was conducted as a cross-sectional survey of public sector medical doctors and nurses in KZN and included those from primary and community health centres and district, regional and tertiary hospitals. As at July 2020, there were 4299 medical doctors and 33422 nursing staff employed by the KZN DOH. In view of the uncertainty of the course of the pandemic and the likely impact on and response from local HCWs, a minimum sample size of 300 participants was planned. This was in keeping with similar studies that had already been conducted and which had sample sizes varying from 69 to 184 participants [17,29]. Convenience and snowball sampling strategies were utilised, with the study being conducted via electronic platforms. Despite the research engaging on multiple platforms and allowing access to the survey for a period of three months, a total of 312 HCWs participated in the study. Eligible participants were English literate. They were required to confirm that they were doctors and nurses employed at public health facilities in KZN during the pandemic. The electronic platforms precluded screening for exclusionary criteria; it was assumed that personnel accessing the electronic survey were physically and mentally capable of participation.

The study questionnaires were created using Survey Monkey, a commercial online survey platform. Information and contact details of available mental health resources and services were included within the survey for all participants to access. The research team engaged in various activities to ensure maximum voluntary participation. The notice of the study as well as the link to the survey questionnaires were circulated via various electronic communication platforms (e-mail/Instant Messaging platforms/social media) used by public sector HCWs. This was posted on the KZN DOH intranet website, which can be accessed by medical and nursing personnel and is available on communal computers within healthcare facilities. The provincial Employee Assistance Program (EAP) coordinator circulated notification of the study and the link to the survey questionnaires to the EAP staff at the various healthcare facilities to encourage eligible persons to participate. The EAP coordinator has communication channels with all KZN DOH healthcare facilities via each facility’s EAP staff members. The South African Medical Association (SAMA) also circulated the notification and link for the study to all their members via e-mail, to raise awareness and facilitate participation in this study.

Ideally, a study of this nature is best undertaken qualitatively. This would have allowed the capturing of individual narratives and allowed for in-depth exploration of the rampant themes of psychological distress that have been associated with the pandemic: fear, anxiety, grief, physical and emotional exhaustion and moral injury [38]. However, the university biomedical research ethics committee imposed strict restrictions on contact research during the Covid-19 pandemic and online interviews were precluded by the lack of hospital e-platforms. Telephonic interviews were not feasible due to the personal cost implications for the participants. Moreover, staff were overwhelmed with coping with the pandemic personally and professionally. Past studies conducted in Africa of a similar nature and topic were also conducted via quantitative methods [27,39]. The sample obtained was recruited with difficulty despite the multiple formal and informal platforms used to advertise it and encourage participation.

This study was approved by the Biomedical Research Ethics Committee (BREC) of the University of KwaZulu-Natal (UKZN) (BREC 00001620/2020) and the KwaZulu-Natal DOH (KZ_202006_021). All study participants provided informed consent electronically.

### Study instruments

The main study outcomes measured were 1) depression, 2) anxiety, 3) traumatic stress related symptoms and 4) subjective perceptions of employer support. Participants completed four questionnaires: a socio-demographic and occupational profile; the DASS-21; the IES-R; and a customised questionnaire relating to perceptions of employer support.

The socio-demographic and occupational profile also contained questions related to perception of risk and degree of involvement in the management of Covid-19 positive patients. Perception of risk was elicited via a closed-ended yes/no question regarding whether the participant felt that they were at an increased risk of contracting Covid-19. If the participant answered in the affirmative, they were then directed to an open-ended question requesting them to cite the reasons for their stated higher perception of risk. Degree of involvement of the HCWs and their respective facility in the assessment and management of patients with Covid-19 was included in the questionnaire. Irrespective of their degree of contact, all medical HCWs were eligible for participation in this study and most facilities eventually had some degree of exposure to Covid-19 positive patients.

The DASS-21 is a 21 item self-report tool designed to measure the emotional states of depression, anxiety and stress, and contains three self-report scales with seven items each that are divided into subscales with similar content. Before interpretation of the scores, the summed numbers in each subscale are multiplied by two (as this is a shortened form of the scale). The cut-off scores for each subscale are: Depression: normal (0–4), mild (5–6), moderate (7–10), severe (11–13), extremely severe (14 or more); Anxiety: normal (0–3), mild (4–5), moderate (6–7), severe (8–9), extremely severe (10 or more); Stress: normal (0–7), mild (8–9), moderate (10–12), severe (13–16), extremely severe (17 or more) [40]. The DASS-21 was utilised in similar previous studies in 2020 [41]. It was also used in other studies locally and shown to be a valid and reliable instrument for research purposes in South Africa [40,42].

The IES-R is a 22 item self-report measure that assesses subjective distress caused by traumatic events. It corresponds to 14 of the 17 Diagnostic and Statistical Manual of Mental Disorders-IV (DSM-IV) symptoms of PTSD, with subscales for intrusion, hyperarousal and avoidance. The IES-R yields a total score ranging from 0 to 88, with a cut-off of 24 being used to define PTSD of clinical concern. The total IES-R score can be graded for severity from normal (0–8), mild (9–25), moderate (26–43) to severe psychological impact (44–48) [43]. The IES-R is one of the most widely used self-report measures within the trauma literature [44]. Review articles reported its use in multiple recent studies relating to the experiences of HCWs during the Covid-19 pandemic [18]. It has been used in other studies locally and shown to be a valid and reliable measure of post-trauma phenomenon in clinical and research settings [35,44,45]. Due to the ongoing nature of the pandemic, the phrasing of the items on the IES-R scale had to be adapted to the present tense. Efforts to contact its authors for permission to do so were unsuccessful. The change of tense did not affect the content validity of the instrument.

Subjective perceptions of employer support were assessed using questions adapted from a study conducted in the USA, which assessed the concerns of HCWs during the Covid-19 pandemic [29]. Their findings were organized into five requests from HCWs to their organization: hear me, protect me, prepare me, support me, and care for me [29]. In our study, we adapted these five requests into six closed-ended questions, with a distinction made between physical and psychological needs. The choice of this measurement was based on the limited studies available that measured perceptions of support, and to enable a comparison.

## Statistical methods

Three analyses were conducted for this investigation: the first summarized the socio-demographic, screening (e.g. depression, anxiety, stress and traumatic stress) and occupational profiles using descriptive statistics; the second summarized the extent of perceived social support during the COVID-19 pandemic using descriptive statistics; the third fitted separate regression models to investigate the roles of socio-demographic factors, occupational profiles and perceived social support against each of the clinical outcomes. A significance of p<0.05 was used for statistical significance testing, with the data being analysed using Stata Version 15.

## Results

### Socio-demographic and occupational profile

A total of 312 HCWs participated; their socio-demographic, occupational and clinical profiles are presented in Table 1. The mean age was 36.6; three-quarters were male (*n* = 234, 75.0%), and more than half were married (*n* = 175, 56.1%) or co-habiting (*n* = 17, 5.5%). The majority (*n* = 214, 72.3%) were medical doctors. Nearly three-quarters (*n* = 178, 64.3%) had 10 years or less of experience post qualification.

**Table 1:** Socio-demographic, clinical and occupational profiles of participants.

		Overall	
		N	%
Gender:			
	Male	234	75.0
	Female	77	24.7
	Non-Binary	1	0.3
Age category:			
	Under 30	79	25.3
	30–59	229	73.4
	60 plus	4	1.3
Race:			
	African	98	31.4
	Coloured	25	8.0
	Indian	152	48.7
	White	32	10.3
	Other	5	1.6
Marital status:			
	Single	106	34.0
	Married	175	56.1
	Co-habiting	17	5.5
	Divorced or widowed	14	4.5
Comorbid medical condition:			
	Hypertension	30	
	Cardiac disease	3	
	Diabetes	12	
	Lung disease	25	
	TB	5	
	HIV	1	
	Endocrine disease	9	
	GI disease	5	
	Musculoskeletal	6	
	Hyperlipidaemia	5	
	Other	13	
Pregnancy:	Yes	6	
Comorbid psychiatric condition:			
	No	273	87.5
	Yes	39	12.5
Occupation role:			
	Medical doctor	214	72.3
	Nursing staff	82	27.7
Years of experience post qualification:			
	Less than 10	178	64.3
	10–19	61	22.0
	More than 20	38	13.7
Healthcare district:			
	eThekwini	196	70.8
	Other	81	29.2
Facility involvement in Covid-19:			
	Yes	270	97.5
	No	7	2.5
Direct contact with Covid-19 positive patients:			
	Yes	238	85.9
	No	39	14.1
Person under Investigation for Covid-19 (HCW):			
	Yes	10	3.7
	No	260	96.3
Tested positive for Covid-19:	Yes	38	14.07
	No	232	85.93
Perception of Covid-19 risk:			
	Yes	248	90.2
	No	27	9.8
Death of a loved one due to Covid-19:			
	Yes	73	27.1
	No	196	72.9
Death of a patient due to Covid-19:			
	Yes	143	53.8
	No	123	46.2

Mean age (and SD) was 36.6 and 9.3 respectively

Among the 312 participants, 120 had pre-existing medical conditions, the most frequent being hypertension (*n* = 30), followed by lung disease *(n* = 25) and diabetes (*n* = 12). A total of 39 participants had pre-existing psychiatric conditions.

Almost all (*n* = 270, 97.5%) reported their facility as being involved in the assessment and/or management of patients with Covid-19, with most (*n* = 238, 85.9%) having had direct contact with Covid-19 positive patients, while 90.2% (*n* = 248) confirmed a higher perception of personal risk, citing a variety of reasons ranging from high-risk exposure, having medical comorbidities and issues relating to Personal Protective Equipment (PPE). Of the total participants, 38 (14.1%) had contracted Covid-19 and 10 were persons under investigation (PUI) for the virus.

Just over one quarter (*n* = 73, 27.1%) had experienced the death of a loved one due to Covid-19. Just over one half (*n* = 143, 53.8%) had experienced the associated death of a patient. No correlation was found between death, either of a loved one or a patient, and measures of distress or feelings of being supported.

### Psychological impact

#### DASS-21

**Fig 1:**
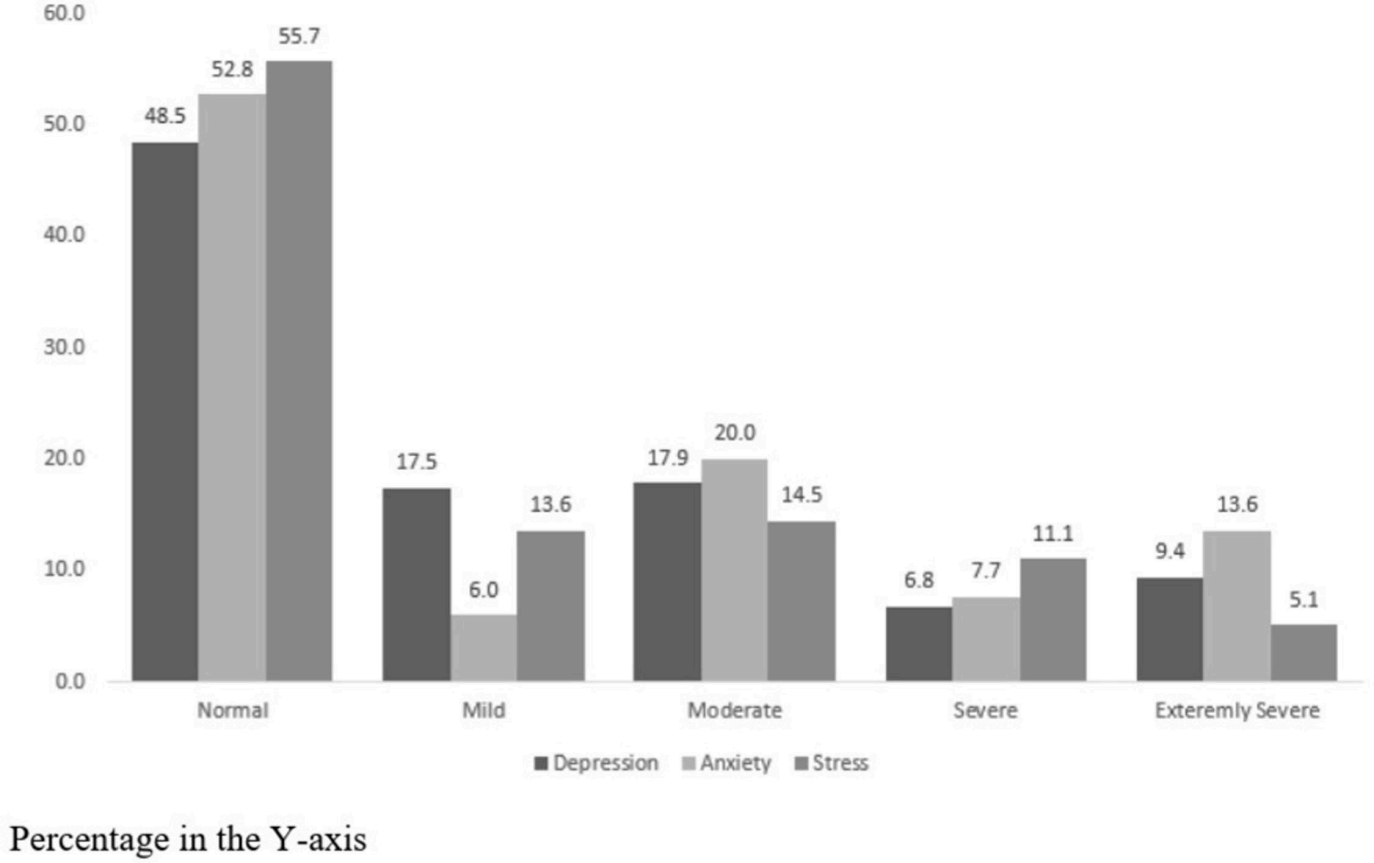
Frequency of symptoms and severity for depression, anxiety and stress by the DASS 21.

The mean DASS-21 score of the sample was 34.8 (standard deviation [SD] 27.4), with depression being the most prevalent of the three symptom categories, followed by anxiety and then stress. According to the DASS-21 (Fig 1), prevalence of study participants with depression, anxiety and stress were 121 (51.5%), 111 (47.2%) and 104 (44.3%) respectively. The number of study participants with combined severe/extremely severe ranges were 38 (16.2%), 50 (21.3%) and 38 (16.2%) for depression, anxiety and stress respectively.

#### IES-R

**Fig 2:**
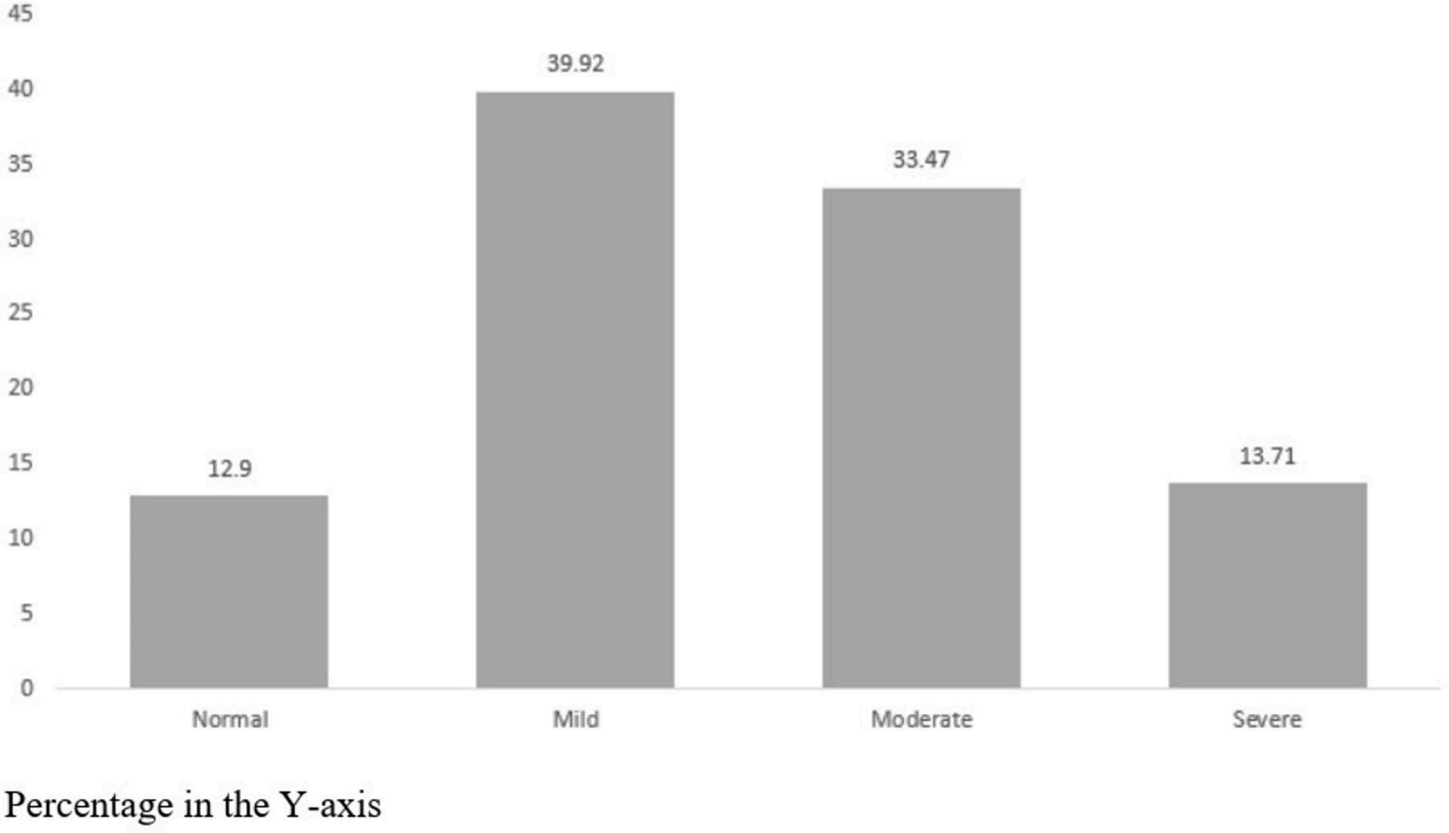
Frequency of symptoms and severity for levels of traumatic stress by the IES-R.

The mean IES-R score was 25.8 (mild psychological impact) (SD 15.6). According to the IES-R, 214 (86.2%) study participants scored between normal to moderate range, with 34 (13.7%) considered to be in the severe range (Fig 2).

### Perceptions of support from employer

Table 2 presents the results related to perceptions of support among the participants.

**Table 2:** Perceptions of support.

		Overall	
		n	%
“Did you feel heard?”			
	Yes	98	37.0
	No	167	63.0
“Did you feel protected?”			
	Yes	143	54.0
	No	122	46.0
“Did you feel prepared?”			
	Yes	107	40.4
	No	158	59.6
“Did you feel physically supported?”			
	Yes	50	18.9
	No	215	81.1
“Did you feel psychologically supported?”			
	Yes	69	26.0
	No	196	74.0
“Did you feel cared for?”			
	Yes	66	24.9
	No	199	75.1

### Regression analyses

The results of the regression analyses are reported in Table 3. The perception of COVID-19 risk (adjusted β = 9.05, p = 0.01) was significantly associated with traumatic stress, as measured by the IES- R. However, the perception of preparedness was significantly associated with lower traumatic stress (adjusted β = -5.53, p = 0.02). Sex (male healthcare workers compared to female) was the only covariate that was significant against other clinical outcomes in anxiety (adjusted β = -3.69, p = 0.01), stress (adjusted β = -4.08, p = 0.01) and traumatic stress (adjusted β = -5.60, p = 0.01).

**Table 3:** Regression results on predictors of mental health.

		Depression			Anxiety			Stress			Traumatic Stress		
		adj β	SE	P	adj β	SE	p	adj β	SE	p	adj β	SE	p
Gender:	[Female]												
	Male	-2.81	1.53	0.07	-3.69	1.39	0.01	-4.08	1.57	0.01	-5.60	2.28	0.01
Age category:	[Under 30]												
	30–59	-2.24	1.69	0.19	-1.66	1.54	0.28	-1.25	1.74	0.47	1.52	2.53	0.55
	60 plus	-7.51	6.27	0.23	-8.56	5.71	0.14	-3.06	6.44	0.64	9.03	9.53	0.34
Marital status:	[Co-habiting]												
	Divorced	1.92	4.61	0.68	1.73	4.20	0.68	-1.50	4.74	0.75	0.96	6.83	0.89
	Married	-4.64	3.44	0.18	-0.97	3.13	0.76	-3.03	3.54	0.39	-2.66	5.00	0.60
	Single	-3.27	3.48	0.35	-0.58	3.17	0.86	-2.50	3.58	0.49	-2.46	5.07	0.63
Occupation:	[Medical]												
	Nursing staff	-1.57	1.64	0.34	0.23	1.49	0.88	-2.84	1.68	0.09	3.45	2.41	0.15
Years’ experience since qualification:	[Less than 10]												
	10–19	1.83	1.74	0.29	1.16	1.58	0.47	1.24	1.78	0.49	-1.44	2.56	0.58
	More than 20	0.81	2.23	0.72	1.57	2.03	0.44	-1.58	2.29	0.49	-6.32	3.30	0.06
Perception of COVID risk:	[No]												
	Yes	3.77	2.24	0.09	3.62	2.04	0.08	4.20	2.30	0.07	9.05	3.27	0.01
“Did you feel prepared?”	[No]												
	Yes	-2.47	1.54	0.11	-1.88	1.40	0.18	-2.06	1.59	0.20	-5.53	2.26	0.02
“Did you feel cared for?”	[No]												
	Yes	-0.52	1.59	0.74	0.35	1.45	0.81	0.03	1.64	0.99	-0.98	2.36	0.68
“Did you feel heard?”	[No]												
	Yes	-1.55	1.54	0.31	-2.14	1.40	0.13	-2.38	1.58	0.13	-0.07	2.26	0.98

Brackets for reference category

## Discussion

In its aim to assess the psychological impact of the COVID-19 pandemic on public sector doctors and nurses in KZN, our study found that at least half of the participants experienced psychological symptoms in the form of depression, anxiety and stress, with one fifth being in the severe range. Additionally, the majority of the participants felt unheard, uncared for and unsupported.

### Psychological impact

Mild to extremely severe symptoms of DAS and traumatic stress were reported by 44.3% to 86.2% of participants; these frequencies exceed findings in similar studies conducted internationally, although there is no available local data against which to compare [18,32,46]. A rapid review of international studies related to the mental health of HCWs during the Covid-19 pandemic was conducted in September 2020 with the prevalence of mental health outcomes varying widely, from 7.0–97.3% for anxiety, 10.6–62.1% for depression, 2.2–93.8% for stress, 3.8–56.6% for PTSD, 8.3–88.4% for insomnia and 21.8–46.3% for burnout [46]. Possible reasons for the higher frequencies of DAS and traumatic stress related symptoms identified in our study could relate to the pre-existing challenges experienced by HCWs in KZN that preceded Covid-19, which were compounded by the superimposed demands of the global pandemic [27,28], as well as the perceived low levels of support from the employer.

A pandemic is classifiable as a traumatic event of exceptional magnitude that transcends the range of normal human experience of exposure to risk of death [18]. This experience can lead to the development of acute stress disorder (ASD) and PTSD [18]. With respect to traumatic stress, 86.2% of our participants were symptomatic and 13.7% considered to be under severe distress, on the IES-R. A review of studies that explored traumatic stress in HCWs in early 2020 reported a prevalence of traumatic stress symptoms (TRSs) of 7.4% - 35% [18]. Pathologic secondary traumatic stress (STS) is defined as “the stress deriving from helping others who are suffering or who have been traumatized” [17]. Under ordinary circumstances, HCWs may be at higher risk of developing pathological secondary traumatisation [47,48]; however, the magnitude, nature and severity of the pandemic combined with its impact on HCW morbidity and mortality globally represent unique stressors which warrant in-depth studies. Such studies should ideally be conducted through qualitative exploration of HCW experiences, both during acute and post-acute phases of the pandemic, given the burden on frontline workers.

### Perception of support

Risk and protective factors for adverse mental health outcomes, in both personal and professional domains, have been identified in the literature. Risk factors include female gender, nursing staff, higher perception of risk, higher degree of Covid-19 exposure, lack of access to PPE, lack of specialised training received, job stress, less job experience, lower levels of organizational support and lower perception of organizational support [11,46]. Protective factors included the perception and provision of psychosocial support, including from the employer, and higher psychological resilience [46]. Higher risk perception is associated with the increased likelihood of developing mental health problems in frontline HCWs [11].

Over 90% (*n* = 248) of participants in our study confirmed a higher perception of Covid-19 risk, citing a variety of reasons, ranging from high-risk exposure, having medical comorbidities and issues relating to PPE. Although no associations were found between DAS and perception of employer support, perception of risk (adjusted β = 9.05, p = 0.01) was significantly associated with greater traumatic stress, as assessed by the IES-R; conversely, the perception of preparedness was significantly associated with lower traumatic stress (adjusted β = -5.53, p = 0.02) with 107 (40.4%) among participants indicating that they felt ‘prepared’. Aligned with the findings of our study, past studies have reported preparedness to be associated with a lower risk of mental health problems [11,32,49]. This highlights the need to formally prepare and train staff for both the biomedical response as well the psychosocial sequelae of a pandemic. While such training can serve as an intervention in preparation for future health crises, it may yet suffice to deconstruct and address ‘preparedness’ as part of an audit of this pandemic and to possibly include ‘preparedness’ in the future curricula of HCWs.

Perception of support was uniformly rated poorly by two thirds to three quarters of participants in our study. Unfortunately, the nature of our study precluded in-depth exploration of the reasons and expectations associated with this finding and there is no available local data which further explores this construct. A qualitative study which, similar to our study, was conducted during the early stages of the pandemic explored the perception of support and its associated factors. HCW needs were summarised as five requests to their organization which could serve as guiding principles in addressing the psychological needs of and support for HCWs during the current pandemic.

The majority of participants in this study (*n* = 296, 95%) reported having access to some form of psychosocial support at their place of employment, although this contrasts starkly with the poorly rated perceptions of support received. While it is assumed that HCWs are expecting extrinsic support, the notion of drawing on inner resources of self-care and self-compassion are also important constructs that must be considered in building a mental health armamentarium in response to stressors of the magnitude of the current pandemic.

While social and workplace support are associated with lower levels of stress and exhaustion, improved immune functioning and higher employment satisfaction [50,51], HCWs are often self-reliant [29]. Many do not exhibit help-seeking behaviours, which may not serve them well in a time of a burgeoning quantitative and qualitative work-load [15], redeployment outside of their area of expertise and the formidable nature of a novel pandemic [29]. Barriers to HCWs accessing available help include poor identification of the symptoms of mental illness in themselves or their colleagues and stoic training environments that normalise stress and distress as inherent or even as requisite components of their identities. Other barriers include stigma against mental illness, rigorous and inflexible work schedules, affordability and confidentiality concerns [52]. The provision of psychological support to HCWs must therefore simultaneously address these multiple and complex barriers in order to be effective.

The protection and support of HCWs, during and after the Covid-19 pandemic, should focus on the development of both physical and psychological immunity. In the context of the aim of this study, the promotion and development of psychological immunity of HCWs is a key component for HCWs. As physical immunity protects individuals from environmental viral infections, similarly, psychological immunity acts as a buffer against environmental stressors [14]. Promoting these ‘antibodies’ in HCWs will act as a deterrent against the development of psychopathology, promote their help-seeking behaviours and empower them towards personal and professional growth [53]. This will have a positive impact on the well-being of HCW and their ability to perform their job effectively.

A systematic review has concluded that there is insufficient evidence from studies carried out during or after disease epidemics and pandemics that can inform the selection of interventions that are beneficial to the mental health of HCWs [24,54]. Alternative sources of evidence, from other healthcare crises, and general evidence about interventions that promote psychological well-being, can therefore be used to inform future planning. Studies conducted during the Ebola outbreak reported that HCWs with effective psychosocial support mechanisms had the lowest symptoms of psychological distress and the best psychological diathesis [55]. Longitudinal research on the specific psychological needs of HCWs will help to generate evidence-based interventions to mitigate the adverse mental health outcomes among HCWs as the Covid-19 pandemic continues into its third year.

Maintaining an adequate healthcare workforce in this crisis requires not only an increased number of HCWs, but also maximising the ability of each clinician to perform optimally in a holistic manner over a protracted time interval [29]. In parallel with the medical and social response to curb the spread and impact of the virus, comprehensive psychosocial support models for HCWs as well as individual- and organization-level interventions directed at this population are equally necessary. A two-pronged model is required that addresses in-pandemic needs and also caters for the post-pandemic aftermath of grief, burnout, depression and post-traumatic stress. Our HCWs may be referred to as the ‘heroes’ [56] of today, but we will still need them tomorrow, physically and mentally healthy [49].

### Limitations

Conducted during an unprecedented health crisis, with participants themselves at the coal-face, our study, unavoidably, has several limitations. There was no specific baseline for DAS and PTS related symptoms in HCWs in this particular area, conducted during the Covid-19 pandemic. The survey was a quantitative study, containing largely closed-ended questions, which limited the depth of exploration of key constructs. Contact research was prohibited during the COVID-19 pandemic, thus confining data collection to electronic platforms, limiting communication about the survey to those who were active online. Telephonic interviews were not feasible due to the personal cost implications for the participants and the challenges of arranging mutually convenient times with the primary researcher who is also a frontline healthcare worker. However, in the context of limited research relevant to this topic within a resource-constrained LMIC setting, we proceeded with this quantitative research study. We view this as a necessary, relevant and well-timed research study that has identified significant findings which can inform and direct future research opportunities and interventions. E-literacy, access to online platforms and connectivity pose significant challenges in our setting for socio-economic reasons, with many public hospitals not having a wireless local area network [28], which may have limited the response rate. We attempted to mitigate some of these challenges by ensuring that the survey was developed to be as user-friendly as possible and arranging for the EAP staff at the healthcare facilities to be able to assist if required. Lockdown conditions, while increasing online activity, also resulted in electronic information overload. This, as well as the ‘additional work’ of completing an online survey may have been a deterrent to fatigued frontline workers. While HCWs may have had differing degrees of involvement, every HCW was employed in a facility which provided Covid-19 care and which could have had a psychological impact on them. We believe that this is actually a strength from the perspective of generalisability of our findings.

## Conclusion and recommendations

High levels of depression, anxiety and post-traumatic stress were detected amongst public sector doctors and nurses employed in KZN during the Covid-19 pandemic in 2020. Additionally, subjective perceptions of employer support were poorly rated. The mental well-being and support of HCWs should be prioritised with an urgent need for more research in this area. The importance of both physical and psychological immunity in HCWs is necessary to protect and preserve their well-being, their ability to function optimally and the robustness of healthcare services as a whole. High levels of DAS and traumatic stress combined with poor perceptions of employer support highlight the need to identify and address the psychosocial support needs and expectations of our HCWs for the duration of the pandemic as well as for the mental health sequelae post-pandemic.

## Supporting information

S1 Table Sociodemographic, clinical and occupational profiles of participants. (PDF)

S2 Table Perceptions of support among participants. (PDF)

S3 Table Regression results on predictors of mental health. (PDF)

S1 Data Dataset. (CSV)

## References

[1] World Health Organisation declares public health emergency for novel coronavirus. Internet. 2020.

[2] Macneil D. Coronavirus has become a pandemic, WHO says. New York Times. www.nytimes.com. 2020.

[3] Centers for Disease Control and Prevention. Introduction to Epidemiology. www.cdc.gov.

[4] Chen Q, Liang M, Li Y, Guo J, Fei D, Wang L, et al. Mental health care for medical staff in China during the COVID-19 outbreak. The Lancet Psychiatry. 2020;7(4):e15–e6. doi: 10.1016/S2215-0366(20)30078-X 32085839 PMC7129426

[5] Vizheh M, Qorbani M, Arzaghi SM, Muhidin S, Javanmard Z, Esmaeili M. The mental health of healthcare workers in the COVID-19 pandemic: A systematic review. J Diabetes Metab Disord. 2020:1–12. doi: 10.1007/s40200-020-00643-9 33134211 PMC7586202

[6] Deng D, Naslund JA. Psychological Impact of Covid-19 Pandemic on Frontline Health Workers in Low- and Middle- Income Countries. Harvard Public Health Review. 2021;2020 Fall; 28. PMC7785092 33409499

[7] Shaukat N, Ali DM, Razzak J. Physical and mental health impacts of COVID-19 on healthcare workers: a scoping review. Int J Emerg Med. 2020;13(1):40. doi: 10.1186/s12245-020-00299-5 32689925 PMC7370263

[8] Karlsson U, Fraenkel CJ. Covid-19: risks to healthcare workers and their families. BMJ. 2020;371:m3944. doi: 10.1136/bmj.m3944 33115772

[9] Update on Covid-19 in The Province. Press release. www.kznonline.gov.za. Published February 2021. Accessed March 2021.

[10] Bekker L-G, Delany-Moretlwe S, Biccard B, duToit L, Lovat LB, Dehbi H-M, et al. Protecting healthcare workers: A critical part of the COVID-19 response. SAMJ: South African Medical Journal. 2020;110:1154–5. doi: 10.7196/SAMJ.2020.v110i12.15048 33403955

[11] Ricci-Cabello I, Meneses-Echavez JF, Serrano-Ripoll MJ, Fraile-Navarro D, de Roque MAF, Moreno GP, et al. Impact of Viral Epidemic Outbreaks on Mental Health of Healthcare Workers: A Rapid Systematic Review. 2020. 10.1016/j.jad.2020.08.034 PMC7443314 32861835

[12] Yaribeygi H, Panahi Y, Sahraei H, Johnston TP, Sahebkar A. The impact of stress on body function: A review. EXCLI J. 2017;16:1057–72. doi: 10.17179/excli2017-480 28900385 PMC5579396

[13] Seiler A, Fagundes CP, Christian LM. The Impact of Everyday Stressors on the Immune System and Health. Stress Challenges and Immunity in Space 2020. p. 71–92.

[14] Gupta T, Nebhinani N. Let’s build the psychological immunity to fight against COVID-19. Indian Journal of Psychiatry. 2020;62(5):601–3. doi: 10.4103/psychiatry.IndianJPsychiatry_420_20 33678854 PMC7909049

[15] Rodriguez BO, Sanchez TL. The Psychosocial Impact of COVID-19 on health care workers. Int Braz J Urol. 2020;46(suppl.1):195–200. doi: 10.1590/S1677-5538.IBJU.2020.S124 32618464 PMC7719993

[16] United N. COVID-19: Mental Illness, a "Parallel Pandemic". www.unric.org. 2021.

[17] Orru G, Marzetti F, Vagheggini G, Conversano C, Miccoli M, Gemignani A, et al. Secondary traumatic stress and burnout in healthcare workers during Covid-19 outbreak. medRxiv. 2020. 10.3390/ijerph18010337 PMC7794988 33466346

[18] Benfante A, Di Tella M, Romeo A, Castelli L. Traumatic Stress in Healthcare Workers During COVID-19 Pandemic: A Review of the Immediate Impact. Front Psychol. 2020;11:569935. doi: 10.3389/fpsyg.2020.569935 33192854 PMC7645025

[19] Chan AO, Huak CY. Psychological impact of the 2003 severe acute respiratory syndrome outbreak on health care workers in a medium size regional general hospital in Singapore. Occup Med (Lond). 2004;54(3):190–6. 15133143 10.1093/occmed/kqh027 PMC7107861

[20] McAlonan GM, Lee A. M., Cheung V., Cheung C., Tsang K. W., Sham P. C., Chua S. E., et al. Immediate and sustained psychological impact of an emerging infectious disease outbreak on health care workers. Canadian journal of psychiatry. Revue canadienne de psychiatrie, 52(4), 241–247. 2007. doi: 10.1177/070674370705200406 17500305

[21] Lee SM, Kang W. S., Cho A. R., Kim T., & Park J. K. Psychological impact of the 2015 MERS outbreak on hospital workers and quarantined hemodialysis patients. Comprehensive psychiatry, 87, 123–127. 2018. doi: 10.1016/j.comppsych.2018.10.003 30343247 PMC7094631

[22] Teo I, Chay J, Cheung YB, Sung SC, Tewani KG, Yeo LF, et al. Healthcare worker stress, anxiety and burnout during the COVID-19 pandemic in Singapore: A 6-month multi-centre prospective study. PLoS One. 2021;16(10):e0258866. doi: 10.1371/journal.pone.0258866 34679110 PMC8535445

[23] Zhang WR, Wang K, Yin L, Zhao WF, Xue Q, Peng M, et al. Mental Health and Psychosocial Problems of Medical Health Workers during the COVID-19 Epidemic in China. Psychother Psychosom. 2020;89(4):242–50. doi: 10.1159/000507639 32272480 PMC7206349

[24] De Kock JH, Latham HA, Leslie SJ, Grindle M, Munoz SA, Ellis L, et al. A rapid review of the impact of COVID-19 on the mental health of healthcare workers: implications for supporting psychological well-being. BMC Public Health. 2021;21(1):104. doi: 10.1186/s12889-020-10070-3 33422039 PMC7794640

[25] Wu P, Fang Y., Guan Z., Fan B., Kong J., Yao Z., et al. The psychological impact of the SARS epidemic on hospital employees in China: exposure, risk perception, and altruistic acceptance of risk. Canadian journal of psychiatry Revue canadienne de psychiatrie, 54(5), 302–311. 2009. doi: 10.1177/070674370905400504 19497162 PMC3780353

[26] Naidoo I, Mabaso M, Moshabela M, Sewpaul R, Reddy S. South African health professionals’state of wellbeing during the emergence of Covid-19. South African Medical Journal. 2020;110. doi: 10.7196/SAMJ.2021.v111i2.15318 33205717

[27] Naidoo T, Tomita A, Paruk S. Burnout, anxiety and depression risk in medical doctors working in KwaZulu-Natal Province, South Africa: Evidence from a multi-site study of resource-constrained government hospitals in a generalised HIV epidemic setting. PLoS One. 2020;15(10):e0239753. doi: 10.1371/journal.pone.0239753 33052921 PMC7556533

[28] Commission PS. Investigation into healthcare facilities in KwaZulu Natal: A special focus on professional ethics. March 2018.

[29] Shanafelt T, Ripp J, Trockel M. Understanding and Addressing Sources of Anxiety Among Health Care Professionals During the COVID-19 Pandemic. JAMA. 2020;323(21):2133–4. doi: 10.1001/jama.2020.5893 32259193

[30] Alnazly E, Khraisat OM, Al-Bashaireh AM, Bryant CL. Anxiety, depression, stress, fear and social support during COVID-19 pandemic among Jordanian healthcare workers. PLoS One. 2021;16(3):e0247679. doi: 10.1371/journal.pone.0247679 33711026 PMC7954309

[31] Rabow MW, Huang CS, White-Hammond GE, Tucker RO. Witnesses and Victims Both: Healthcare Workers and Grief in the Time of COVID-19. J Pain Symptom Manage. 2021. doi: 10.1016/j.jpainsymman.2021.01.139 33556494 PMC7864782

[32] Robertson LJ, Maposa I, Somaroo H, Johnson O. Mental health of healthcare workers during the COVID-19 outbreak: A rapid scoping review to inform provincial guidelines in South Africa. S Afr Med J. 2020;110(10):1010–9. doi: 10.7196/SAMJ.2020.v110i10.15022 33205731

[33] Ding Y QJ, Yu X, Wang S. The mediating effects of burnout on the relationship between anxiety symptoms and occupational stress among community healthcare workers in China: a cross-sectional study. PLoS One. 2014;9(9):e107130. doi: 10.1371/journal.pone.0107130 25211025 PMC4161428

[34] Ruotsalainen JH, Verbeek JH, Marine A, Serra C. Preventing occupational stress in healthcare workers. Cochrane Database Syst Rev. 2014(11):CD002892. doi: 10.1002/14651858.CD002892.pub3 25391582

[35] Rana W, Mukhtar S, Mukhtar S. Mental health of medical workers in Pakistan during the pandemic COVID-19 outbreak. Asian J Psychiatr. 2020;51:102080. doi: 10.1016/j.ajp.2020.102080 32283512 PMC7139243

[36] DOH-KZN. Covid 19 Mental Health Toolkit. www.kznhealth.gov.za. 2020.

[37] NICD-NHLS. An update on Covid 19 outbreak in South Africa The first and second wave of cases in South Africa. www.nicd.ac.za. January 2021.

[38] Tremblay S, Castiglione S, Audet L-A, Desmarais M, Horace M, Peláez S. Conducting Qualitative Research to Respond to COVID-19 Challenges: Reflections for the Present and Beyond. International Journal of Qualitative Methods. 2021;20.

[39] Migisha R, Ario AR, Kwesiga B, Bulage L, Kadobera D, Kabwama SN, et al. Risk perception and psychological state of healthcare workers in referral hospitals during the early phase of the COVID-19 pandemic, Uganda. BMC Psychol. 2021;9(1):195. doi: 10.1186/s40359-021-00706-3 34920763 PMC8678424

[40] Tshabalala SJ, Tomita A, Ramlall S. Depression, anxiety and stress symptoms in patients presenting with dyspepsia at a regional hospital in KwaZulu-Natal province. S Afr J Psychiatr. 2019;25:1382. doi: 10.4102/sajpsychiatry.v25i0.1382 31745439 PMC6852706

[41] Shah SMA, Mohammad D, Qureshi MFH, Abbas MZ, Aleem S. Prevalence, Psychological Responses and Associated Correlates of Depression, Anxiety and Stress in a Global Population, During the Coronavirus Disease (COVID-19) Pandemic. Community Ment Health J. 2021;57(1):101–10. doi: 10.1007/s10597-020-00728-y 33108569 PMC7590908

[42] Dreyer Z, Henn C, Hill C. Validation of the Depression Anxiety Stress Scale-21 (DASS-21) in a non-clinical sample of South African working adults. Journal of Psychology in Africa. 2019;29(4):346–53.

[43] Lai J, Ma S, Wang Y, Cai Z, Hu J, Wei N, et al. Factors Associated With Mental Health Outcomes Among Health Care Workers Exposed to Coronavirus Disease 2019. JAMA Netw Open. 2020;3(3):e203976. doi: 10.1001/jamanetworkopen.2020.3976 32202646 PMC7090843

[44] Beck JG, Grant DM, Read JP, Clapp JD, Coffey SF, Miller LM, et al. The impact of event scale-revised: psychometric properties in a sample of motor vehicle accident survivors. J Anxiety Disord. 2008;22(2):187–98. doi: 10.1016/j.janxdis.2007.02.007 17369016 PMC2259224

[45] Vassar MKK HW, Hale H. A meta analysis of coefficient alpha for the Impact of Event Scales a reliability generalization study South African Journal of Psychology 2011;41(1):6–16.

[46] Major A, Hlubocky FJ. Mental health of healthcare workers during the Covid-19 pandemic and evidence based frameworks for mitigation: A rapid review. medRxiv. 2021.

[47] Beck CT. Secondary traumatic stress in nurses: a systematic review. Archives of psychiatric nursing, 25(1), 1–10 2011. doi: 10.1016/j.apnu.2010.05.005 21251596

[48] Ogińska-Bulik N GP, Michalska P, Kędra E Prevalence and predictors of secondary traumatic stress symptoms in health care professionals working with trauma victims: A cross-sectional study. PLoS ONE 16(2): e0247596. 2021. doi: 10.1371/journal.pone.0247596 33621248 PMC7901735

[49] Greenberg N, Docherty M, Gnanapragasam S, Wessely S. Managing mental health challenges faced by healthcare workers during covid-19 pandemic. BMJ. 2020;368:m1211. doi: 10.1136/bmj.m1211 32217624

[50] Ledikwe JH, Kleinman NJ, Mpho M, Mothibedi H, Mawandia S, Semo BW, et al. Associations between healthcare worker participation in workplace wellness activities and job satisfaction, occupational stress and burnout: a cross-sectional study in Botswana. BMJ Open. 2018;8(3):e018492. doi: 10.1136/bmjopen-2017-018492 29549200 PMC5857656

[51] Uchino BN, Vaughn AA, Carlisle M, Birmingham W. Social support and immunity. The Oxford handbook of psychoneuroimmunology. Oxford library of psychology. New York, NY, US: Oxford University Press; 2012. p. 214–33.

[52] Kalmoe MC, Chapman MB, Gold JA, Giedinghagen AM. Physician Suicide: A Call to Action Missouri Medicine. 2019;May-June(116 (3)):211–6. 31527944 PMC6690303

[53] Jaiswal A, Singh T, Arya YK. "Psychological Antibodies" to Safeguard Frontline Healthcare Warriors Mental Health Against COVID-19 Pandemic-Related Psychopathology. Front Psychiatry. 2020;11:590160. doi: 10.3389/fpsyt.2020.590160 33391053 PMC7775359

[54] Pollock A, Campbell P, Cheyne J, Cowie J, Davis B, McCallum J, et al. Interventions to support the resilience and mental health of frontline health and social care professionals during and after a disease outbreak, epidemic or pandemic: a mixed methods systematic review. Cochrane Database Syst Rev. 2020;11:CD013779. doi: 10.1002/14651858.CD013779 33150970 PMC8226433

[55] Dong Ji Y-JJ, Duan Xue-Zhang, Li Wen-Gang, Sun Zhi-Qiang, Xue-Ai Song Y-HM, Tang Hong-Mei, et al. Prevalence of psychological symptoms among Ebola survivors and healthcare workers during the 2014–2015 Ebola outbreak in Sierra Leone: a cross-sectional study. Oncotarget, 2017, Vol 8, (No 8), pp: 12784–12791. 2017. doi: 10.18632/oncotarget.14498 28061463 PMC5355054

[56] DOH-KZN. From The Frontlines: An appeal from a healthcare hero. www.sacoronavirus.co.za. 2021.

