# Supplementary material for: ‘Unheard,’ ‘uncared for’ and ‘unsupported’: The mental health impact of Covid -19 on healthcare workers in KwaZulu-Natal Province, South Africa
Source: PLoS One. 2022 May 4;17(5):e0266008. doi: 10.1371/journal.pone.0266008 (PMC9067674; doi:10.1371/journal.pone.0266008)
Supplement: S1 Table — (PDF) [file pone.0266008.s001.pdf]

Table 1: Socio-demographic, clinical and occupational profiles

		Overall	
		N	%
Gender:			
	Male	234	75.0
	Female	77	24.7
	Non-Binary	1	0.3
Age category:			
	Under 30	79	25.3
	30-59	229	73.4
	60 plus	4	1.3
Race:			
	African	98	31.4
	Coloured	25	8.0
	Indian	152	48.7
	White	32	10.3
	Other	5	1.6
Marital status:			
	Single	106	34.0
	Married	175	56.1
	Co-habiting	17	5.5
	Divorced or widowed	14	4.5
Comorbid medical condition:			
	Hypertension	30	
	Cardiac disease	3	
	Diabetes	12	
	Lung disease	25	
	TB	5	
	HIV	1	
	Endocrine disease	9	
	GI disease	5	
	Musculoskeletal	6	
	Hyperlipidaemia	5	
	Other	13	
Pregnancy:	Yes	6	
Comorbid psychiatric condition:			
	No	273	87.5
	Yes	39	12.5
Occupation role:			
	Medical doctor	214	72.3
	Nursing staff	82	27.7
Years of experience post qualification:			
	Less than 10	178	64.3
	10-19	61	22.0
	More than 20	38	13.7
Healthcare district:			
	eThekwini	196	70.8
	Other	81	29.2
Facility involvement in Covid-19:			
	Yes	270	97.5
	No	7	2.5
Direct contact with Covid-19 positive patients:			
	Yes	238	85.9
	No	39	14.1
Person under Investigation for Covid-19 (HCW):			
	Yes	10	3.7
	No	260	96.3
Tested positive for Covid-19:	Yes	38	14.07
	No	232	85.93
Perception of Covid-19 risk:			
	Yes	248	90.2
	No	27	9.8
Death of a loved one due to Covid-19:			
	Yes	73	27.1
	No	196	72.9
Death of a patient due to Covid-19:			
	Yes	143	53.8
	No	123	46.2

Mean age (and SD) was 36.6 and 9.3 respectively
