# Supplementary material for: ‘Unheard,’ ‘uncared for’ and ‘unsupported’: The mental health impact of Covid -19 on healthcare workers in KwaZulu-Natal Province, South Africa
Source: PLoS One. 2022 May 4;17(5):e0266008. doi: 10.1371/journal.pone.0266008 (PMC9067674; doi:10.1371/journal.pone.0266008)
Supplement: S2 Table — (PDF) [file pone.0266008.s002.pdf]

Table 2
Perceptions of support

		Overall	
		n	%
"Did you feel heard?"			
	Yes	98	37.0
	No	167	63.0
"Did you feel protected?"			
	Yes	143	54.0
	No	122	46.0
"Did you feel prepared?"			
	Yes	107	40.4
	No	158	59.6
"Did you feel physically supported?"			
	Yes	50	18.9
	No	215	81.1
"Did you feel psychologically supported?"			
	Yes	69	26.0
	No	196	74.0
"Did you feel cared for?"			
	Yes	66	24.9
	No	199	75.1
