# Supplementary material for: ‘Unheard,’ ‘uncared for’ and ‘unsupported’: The mental health impact of Covid -19 on healthcare workers in KwaZulu-Natal Province, South Africa
Source: PLoS One. 2022 May 4;17(5):e0266008. doi: 10.1371/journal.pone.0266008 (PMC9067674; doi:10.1371/journal.pone.0266008)
Supplement: S3 Table — (PDF) [file pone.0266008.s003.pdf]

**Table 3**
Regression results

| | | Depression | | | Anxiety | | | Stress | | | Traumatic Stress | | |
|---|---|---|---|---|---|---|---|---|---|---|---|---|---|
| | | adj β | SE | P | adj β | SE | p | adj β | SE | p | adj β | SE | p |
| Gender: | [Female] | | | | | | | | | | | | |
| | Male | -2.81 | 1.53 | 0.07 | -3.69 | 1.39 | 0.01 | -4.08 | 1.57 | 0.01 | -5.60 | 2.28 | 0.01 |
| Age category: | [Under 30] | | | | | | | | | | | | |
| | 30-59 | -2.24 | 1.69 | 0.19 | -1.66 | 1.54 | 0.28 | -1.25 | 1.74 | 0.47 | 1.52 | 2.53 | 0.55 |
| | 60 plus | -7.51 | 6.27 | 0.23 | -8.56 | 5.71 | 0.14 | -3.06 | 6.44 | 0.64 | 9.03 | 9.53 | 0.34 |
| Marital status: | [Co-habiting] | | | | | | | | | | | | |
| | Divorced | 1.92 | 4.61 | 0.68 | 1.73 | 4.20 | 0.68 | -1.50 | 4.74 | 0.75 | 0.96 | 6.83 | 0.89 |
| | Married | -4.64 | 3.44 | 0.18 | -0.97 | 3.13 | 0.76 | -3.03 | 3.54 | 0.39 | -2.66 | 5.00 | 0.60 |
| | Single | -3.27 | 3.48 | 0.35 | -0.58 | 3.17 | 0.86 | -2.50 | 3.58 | 0.49 | -2.46 | 5.07 | 0.63 |
| Occupation: | [Medical] | | | | | | | | | | | | |
| | Nursing staff | -1.57 | 1.64 | 0.34 | 0.23 | 1.49 | 0.88 | -2.84 | 1.68 | 0.09 | 3.45 | 2.41 | 0.15 |
| Years' experience since qualification: | [Less than 10] | | | | | | | | | | | | |
| | 10-19 | 1.83 | 1.74 | 0.29 | 1.16 | 1.58 | 0.47 | 1.24 | 1.78 | 0.49 | -1.44 | 2.56 | 0.58 |
| | More than 20 | 0.81 | 2.23 | 0.72 | 1.57 | 2.03 | 0.44 | -1.58 | 2.29 | 0.49 | -6.32 | 3.30 | 0.06 |
| Perception of COVID risk: | [No] | | | | | | | | | | | | |
| | Yes | 3.77 | 2.24 | 0.09 | 3.62 | 2.04 | 0.08 | 4.20 | 2.30 | 0.07 | 9.05 | 3.27 | 0.01 |
| "Did you feel prepared?" | [No] | | | | | | | | | | | | |
| | Yes | -2.47 | 1.54 | 0.11 | -1.88 | 1.40 | 0.18 | -2.06 | 1.59 | 0.20 | -5.53 | 2.26 | 0.02 |
| "Did you feel cared for?" | [No] | | | | | | | | | | | | |
| | Yes | -0.52 | 1.59 | 0.74 | 0.35 | 1.45 | 0.81 | 0.03 | 1.64 | 0.99 | -0.98 | 2.36 | 0.68 |
| "Did you feel heard?" | [No] | | | | | | | | | | | | |
| | Yes | -1.55 | 1.54 | 0.31 | -2.14 | 1.40 | 0.13 | -2.38 | 1.58 | 0.13 | -0.07 | 2.26 | 0.98 |

Brackets for reference category
